# Human Activity Recognition Based on Embedded Sensor Data Fusion for the Internet of Healthcare Things

**DOI:** 10.3390/healthcare10061084

**Published:** 2022-06-10

**Authors:** Mohamed E. Issa, Ahmed M. Helmi, Mohammed A. A. Al-Qaness, Abdelghani Dahou, Mohamed Abd Elaziz, Robertas Damaševičius

**Affiliations:** 1 Computer and Systems Engineering Department, Faculty of Engineering, Zagazig University, Zagazig 44519, Egypt; mamohamedali@eng.zu.edu.eg (M.E.I.); amhm162@gmail.com (A.M.H.); 2 College of Engineering and Information Technology, Buraydah Private Colleges, Buraydah 51418, Saudi Arabia; 3 State Key Laboratory for Information Engineering in Surveying, Mapping and Remote Sensing, Wuhan University, Wuhan 430079, China; 4 Faculty of Engineering, Sana’a University, Sana’a 12544, Yemen; 5 LDDI Laboratory, Faculty of Science and Technology, University of Ahmed DRAIA, Adrar 01000, Algeria; dahou.abdghani@univ-adrar.edu.dz; 6 Faculty of Computer Science and Engineering, Galala University, Suez 435611, Egypt; abd_el_aziz_m@yahoo.com; 7 Artificial Intelligence Research Center (AIRC), College of Engineering and Information Technology, Ajman University, Ajman 346, United Arab Emirates; 8 Department of Mathematics, Faculty of Science, Zagazig University, Zagazig 44519, Egypt; 9 Department of Applied Informatics, Vytautas Magnus University, 44404 Kaunas, Lithuania

**Keywords:** Internet of Healthcare Things, human activity recognition, smart technologies for healthcare, m-Health, mobile devices, digital healthcare

## Abstract

Nowadays, the emerging information technologies in smart handheld devices are motivating the research community to make use of embedded sensors in such devices for healthcare purposes. In particular, inertial measurement sensors such as accelerometers and gyroscopes embedded in smartphones and smartwatches can provide sensory data fusion for human activities and gestures. Thus, the concepts of the Internet of Healthcare Things (IoHT) paradigm can be applied to handle such sensory data and maximize the benefits of collecting and analyzing them. The application areas contain but are not restricted to the rehabilitation of elderly people, fall detection, smoking control, sportive exercises, and monitoring of daily life activities. In this work, a public dataset collected using two smartphones (in pocket and wrist positions) is considered for IoHT applications. Three-dimensional inertia signals of thirteen timestamped human activities such as Walking, Walking Upstairs, Walking Downstairs, Writing, Smoking, and others are registered. Here, an efficient human activity recognition (HAR) model is presented based on efficient handcrafted features and Random Forest as a classifier. Simulation results ensure the superiority of the applied model over others introduced in the literature for the same dataset. Moreover, different approaches to evaluating such models are considered, as well as implementation issues. The accuracy of the current model reaches 98.7% on average. The current model performance is also verified using the WISDM v1 dataset.

## 1. Introduction

### 1.1. Motivation

Smart solutions for Internet of Healthcare Things (IoHT) [1], also known as Healthcare Internet of Things [2], Internet of Medical Things [3], or Medical Internet of Things [4], systems have extensively emerged since the Industry 4.0 revolution [5], making use of digital devices, in particular wearable sensors and smart handheld devices. In the new phase of the industrial revolution, termed Industry 5.0, collaborative interaction between machines and people is coming back to the forefront [6]. Unlike aiming to find the best ways to connect devices together—in the first place—which was a goal of Industry 4.0, there is great interest in moving toward personalization in Industry 5.0. This means that creative thinking and smart usage of the entities of smart systems are expected to increase the productivity and benefits of emerging IoT-based solutions [7]. The guidelines of Industry 5.0—under the umbrella of IoT—open up a new window to the development and enhancement of existing smart IoHT systems, in particular, during present-day circumstances, such as the spread of COVID-19, and ehealth and telehealth services can be provided without in-person visits [8,9], while decision support provided by artificial intelligence methods can facilitate doctors’ decisions [10]. Numerous applications are categorized under IoHT applications. For example, indoor localization and IoT applications inside smart buildings such as keeping social distances have been used since the COVID-19 pandemic began [11]. In addition, such applications are used for traditional tasks such as the monitoring of daily life activity [5,12,13], fall detection [14] and assisted living [15,16,17,18], bad habits (such as smoking) detection and control [19], monitoring of industrial workers’ activity [20], monitoring the heart rate of vehicle drivers [21], using wearable sensors to monitor heart activity [22,23], mHealth Apps for Self-Management [24], gait detection for people with Parkinson’s disease [25,26], and many others.

The implementation of IoHT systems starts with data acquisition, followed by a preprocessing and feature-extraction phase, and finally arrives at the decision-making stage. Most known approaches in the literature can be categorized as video-based, WiFi-based, and sensory-based. Video-based human activity monitoring approaches may provide rich information via videos and images for indoor activities when there are no ad hoc cameras in outdoor environments such as walking tracks, parks, traditional malls, and swimming pools. Conversely, both wearable sensors and smart handheld devices are very suitable for the environment-invariant Human Activity Recognition (HAR) models. Another concern is that maintaining the privacy of individuals is questionable in vision-based approaches [27], while dealing with data fusion from sensors presents no such compromise. However, WiFi-based recognition of activities of daily life [28,29,30] has the advantage of using the fixed WiFi devices, but such approach has no applicability in outdoor environments.

A great interest is devoted to employing wearable sensors (e.g., accelerometer units), embedded sensors in smart devices (e.g., accelerometer, gyroscope, and magnetometer), and Kinect sensors [31] to develop HAR models [12,32]. Currently, smart devices such as smartphones and smartwatches are receiving much attention in such IoHT applications for obvious reasons [5]. On the other hand, a special-purpose real-time health monitoring device may have concerns regarding the efficient implementation in terms of power consumption [33]. When data acquisition is performed through many sensors and/or devices, there is a need for a suitable IoT framework to be able to move to the preprocessing stage. In preprocessing stage, the tri-axial activity signals registered by the sensors usually first need noise filtration, then segmentation in window length that ranges from <1 to 30 s [5,34] with more focus on reasonable small lengths (e.g., 2–10 s) in order to simulate real-time situations. Furthermore, feature extraction can follow the traditional approach of handcrafting a set of fine features selected in the time domain (mean, standard deviation, min, max, Pearson coefficients, etc.) and the frequency domain (energy, entropy, FFT coefficients, etc.), or they may follow the modern trend of deep learning networks [16,34]. In the latter approach, features are implicitly extracted as the encodings of hidden layers of the network, while outer layers such as fully connected layers together with softmax layer are responsible for the decision-making (i.e., classification and recognition). Following the feature engineering approach, the Random Forest (RF) algorithm [35], Multilayer Perceptron (MLP) [36] (one variant of artificial neural networks), Support Vector Machines (SVMs) [37], and Naive Bayes (NB) [38] are among the well-known shallow classifiers.

However, deep learning models perform well for many available human activity datasets in the literature [34], but the RF algorithm, for example, performs better than a single LSTM classifier for a specific dataset addressed in [16]. In addition, the recent studies in [17,39,40,41] in IoT applications depend on shallow classifiers. Recently, hybrid ensemble approaches that make use of shallow classifiers in addition to deep convolutional layers are significantly bullish [28].

The limitation of existing approaches concerning a dataset collected by two smartphone units (in pocket and wrist positions) of human activities and gestures introduced by Shoaib et al. [42] motivates improving the state-of-the-art results. In this paper, an interesting and challenging dataset of thirteen activities is addressed. Activities are divided into two groups: the first group consists of hand gestures such as eating, smoking, drinking coffee, typing, and writing, and the other group consists of biking, jogging, standing, sitting, walking, walking upstairs, and walking downstairs. As a classification problem, the whole dataset is handled at a time in the training and testing processes. Using a feature set that is adequate to sensors’ positions on the human body, an impartial comparison between the aforementioned shallow classifiers is conducted. The RF algorithm shows outstanding performance compared to previous models in the literature according to both subject-dependent and stratified k-fold cross-validation evaluation metrics. Furthermore, for testing the model generalization, another dataset, namely WISDM v1 [43], is used to examine the applied model performance.

### 1.2. Related Work

In the literature, numerous human activity datasets were collected from smartphones and/or smartwatches, e.g., WISDM v1 and v2, UCI–HAR, and UniMiB SHAR; see the survey by Demrozi et al. [44] for complete details. Shoaib et al. published a public dataset in [42] using two smartphone units. Below, we shed light on some closely related studies that addressed this dataset. In [42], a simple feature set of mean, standard deviation, median, min, max, semi-quartile, and the sum of the first ten FFT coefficients were extracted from each sensor stream, and the magnitude of its 3-dimensional signal was applied to the NB classifier. Since the readings of the accelerometer, linear accelerometer, gyroscope, and magnetometer sensors in both smartphones were registered, the focus in [42] was to evaluate the combination of sensors and device positions on the body, besides determining the effect of the window length from 2 to 30 s. The accelerometer and the gyroscope from both devices’ positions gave the best performance. Baldominos et al. [45] performed a comparative study between different machine learning techniques (deep and shallow). Readings of the four sensors mentioned above were used. For shallow techniques, handcrafted features such as the mean and the standard deviation of raw signals and skewness, kurtosis, and the lower and upper quartiles of real coefficients of FFT of each dimension were obtained. The ensemble of randomized decision trees (ET) outperformed both shallow classifiers such as RF, MLP, NB, and K-nearest neighbors and convolutional neural networks (CNN). Alo et al. [46] examined two deep learning models, namely deep-stacked autoencoders (DSAE) and deep belief neural networks (DBNN). Only signals of the accelerometer are considered in both devices. Besides raw signals, the magnitude vector and the vectors of pitch and roll angles are used for training the models. The DSAE showed notable performance over both DBNN and the shallow classifiers (with the time-domain features in [42]) such as SVM, NB, and linear discriminant analysis. There are also deep learning models proposed for HAR using wearable sensors. For example, in [47], a combination of long short-term memory (LSTM) and a conventional neural network (CNN) was proposed to solve the HAR problem. In [48], a new HAR model was developed based on convolutional and LSTM recurrent units. In [49], a new model called iSPLInception was developed based on the Inception-ResNet framework from Google. It showed acceptable performance using different HAR datasets. In [50], the authors studied the applications of several deep learning methods. They found that the hybrid CNN-BiGRU showed the best results. Among the aforementioned studies, stratified k-fold evaluation criteria were applied by Shoaib et al. [42], while dataset samples were divided into train/test sets with a subject-dependent measure in [45,46]. Moreover, there is a variance between the different studies about the most suitable sensors for this task. Finally, there is some confusion about the superiority of conventional machine learning approaches versus deep learning models for this specific dataset.

To solve such conflicts, this paper proposes an individual model that proves superior according to both evaluation criteria. In addition, an impartial comparison between previous approaches and the current one has been performed.

### 1.3. Contribution of Current Work

- Presenting a light human-activity-recognition system using wearable sensors.
- Implementing a robust real-time model based on the Random Forest algorithm that outperforms other known classifiers and deep learning models.
- Handling a complex dataset of thirteen different human activities and gestures and improving the state-of-the-art results according to both subject-dependent and stratified k-fold cross-validation measures and using a different dataset, namely WISDM v1, for verifying model performance.
- Conducting sensitivity analysis for the applied model parameters (Random Forest size and depth).

### 1.4. Paper Organization

This document is organized as follows: Section 2 introduces the applied IoHT system framework. Section 3 presents the experimental results within the discussion. Section 4 handles the effect of important parameters on model performance. Section 5 provides a comparison with previous related studies. A different dataset is used to verify model performance in Section 6. The discussion of obtained results is given in Section 7. Section 8 includes conclusions, limitations, and future extensions of this work.

## 2. The Applied Approach

### 2.1. Dataset

Table 1 presents the generic information of dataset addressed here. Activity signals were recorded at a frequency of 50 Hz from the accelerometer, linear accelerometer, gyroscope, and magnetometer sensors of two Samsung Galaxy S2 smartphones. One device was put in the right pocket, and the other was placed on the right wrist. Ten subjects were asked to perform thirteen activities following a protocol; see Table 2 for the duration of each activity performed for each subject. This data set comprises six activities involving hand gestures, namely eating, smoking, drinking coffee, typing, and writing, and seven activities involving full-body motions, namely biking, jogging, standing, sitting, walking, walking upstairs, and walking downstairs. The total number of observations was 1,170,000. Activity signals were successfully registered, and there were no missing values. More details about the settings of collecting activities can be reviewed in [42].

### 2.2. Sensory Data Processing

**Figure 1:**
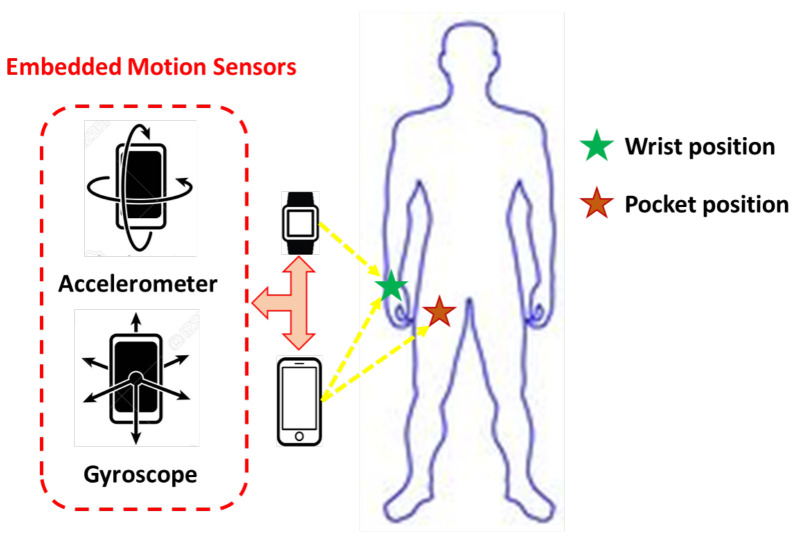
Activity signal acquisition from handheld smart devices.

**Figure 2:**
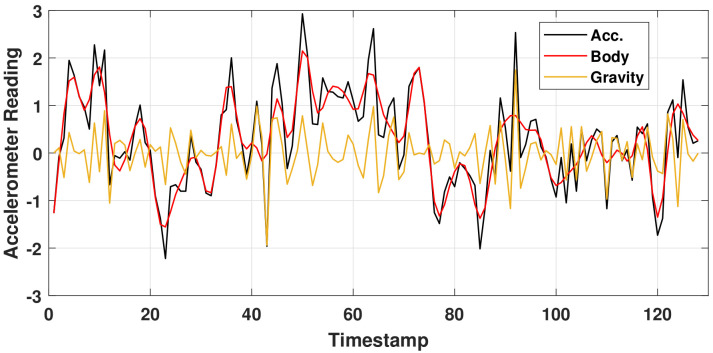
Accelerometer signal separation into body and gravity components using the Butterworth filter with a corner frequency of 20 Hz.

**Figure 3:**
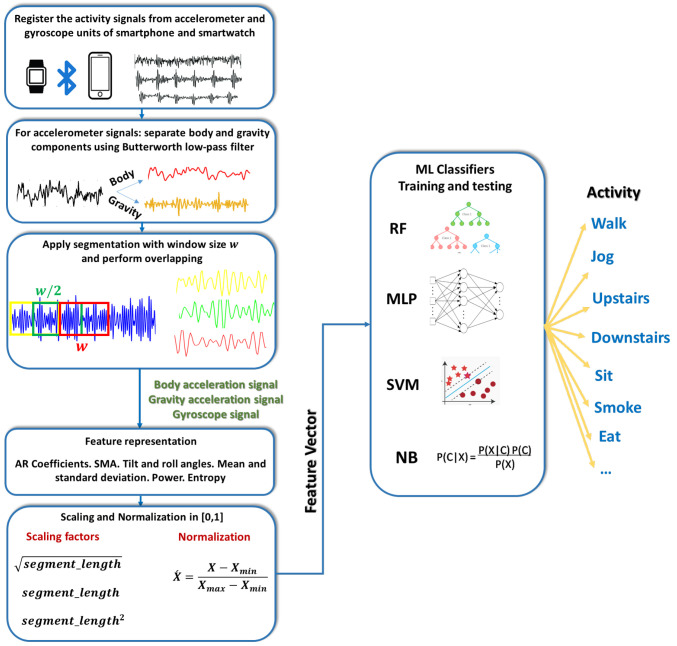
The composition of the applied IoHT system.

The applied model makes use of the readings of accelerometer and gyroscope sensors, where the acceleration and angular velocity of body limbs are sufficient for characterizing the activities performed. This point of view coincides with the well-known study of Anguita et al. [51]. Figure 1 clarifies the sensors’ positions on the human body in order to acquire activity signals. Figure 2 shows the signal separation into body and gravity components using the Butterworth filter. Figure 3 presents the IoHT framework applied here. When applying the model, it is suggested to connect devices through Bluetooth technology. Then, the processing takes place at one central point (i.e., smartphone) as shown in Figure 3.

*Activity Signal Preprocessing*. According to previous studies, e.g., [51,52,53], it is preferred separate body and gravity components of accelerometer signals using, for example, a fourth-order Butterworth low-pass filter with a corner frequency of 20 Hz to filter out the body-acceleration component, since signals were collected at 50 Hz–. For real-time considerations, signals were segmented using a window length of 2.56 s (i.e., 128 data points) with an overlap of 50% [51]. Figure 2 presents an illustrating example of acceleration signal separation for the walking activity in a time interval of 2.56 s. Thus, there is a fusion of six time-series signals: body acceleration, gravity acceleration, and gyroscope readings of both devices.

*Feature Representation*. The features for smartphone-based activity signals (with the numerical participation in the feature vector in parenthesis) are listed as follows:

- (F1-12) Mean and standard deviation (STD) of each of the acceleration signal (AS) and its jerk signal (JS)
- (F13-24) Autoregressive (AR) model coefficients for AS
- (F25) Signal magnitude area (SMA)
- (F26) Tilt angle (TA)
- (F27-30) Roll angle (RA) Equation (1): mean, STD, entropy of JS, and power
- (F31) Angle of x-component of AS Equation (2)
- (F32-34) Entropy of JS
- (F35-37) Power of AS

(1) $$\mathrm{Roll}\;\mathrm{angle}=arctan(-BA_{z},-BA_{y})$$

where $BA_{y}$ and $BA_{z}$ are body acceleration in *y* and *z* dimensions, respectively.

(2) $$\begin{array}{c} \mathrm{Angle}\;\mathrm{of}\;x-\mathrm{component}\;\mathrm{of}\;\mathrm{AS}=real\left(arccos\left(max\left(min\left(\frac{B_{x}\cdot G_{m}}{\left|\right|B_{x}\left||*|\right|G_{m}\left|\right|},1\right),-1\right)\right)\right) \end{array}$$

where the only real part of the resulting quantity is used; $B_{x}$ and $G_{m}$ are body acceleration in the x-axis and the mean of gravity component in 3D, respectively; and the denominator represents the multiplication of the 2-norm of each vector. For the rest of the features, the readers can review [51]. Such a feature set is sensitive to body kinematics (e.g., wrist and leg motion in action). Thus, the 3D signals of each of the four operating sensors are represented by 37 features. Furthermore, combining the extracted features results in a 222-dimensional feature vector where the separation of body and gravity components of the accelerometer is performed.

*Scaling and Normalization.* The numerical values of the feature vector have a great variance in magnitude; e.g., SMA can reach a value that is a few hundred times that of the power of AS and the STD of acceleration JS. In order to eliminate the negative effect on the classification task, scaling is performed in terms of the segment length (slen). The coefficients of the AR model, TA, mean, and STD of AS, mean of JS, mean of RA, and power of RA are scaled by $\sqrt{slen}$, while the angle of the x-component of AS is scaled by slen, and finally the scaling factor $slen^{2}$ is applied for SMA. The rest of the features are used without scaling. This treatment is heuristically examined. After that, the whole feature vector is normalized in [0,1] as illustrated in Figure 3.

*Classification Layer.* Commonly applied classification algorithms in human-activity-recognition tasks are referred to here as RF, MLP, SVM, adn NB. RF [35] is a voting-based classifier where a decision tree is created for each sample inside a random subset of features. Then, the decision is taken for the sake of the class that is the most voted for. Thus, the most important parameters of the RF classifier are the number of decision trees and the maximum depth of the tree. MLP [36] contains interconnected processing units called neurons in one or more layers. Each neuron is characterized by its activation function, that is, a function of the weights of the preceding layer. The training algorithm, which is responsible for finding the best weights, plays a vital role in the network performance. In addition, the number of layers, number of neurons, and type of activation function are the most important parameters for the MLP. SVM [37] depends on finding the best hyperplanes that achieve the maximal margin between the nearest examples in high-dimensional spaces of two different classes. For a multiclass problem, n∗(n−1)/2 binary SVM models are generated to distinguish *n* classes. NB [38] is a simple classifier that makes use of Bayes’ rule to determine the class with the highest posterior probability.

## 3. Experimental Results and Analysis

### 3.1. Setup

Well-known machine learning (ML) classifiers in the IoT area, namely RF, MLP, SVM, and NB, are examined in an impartial comparison in order to clarify the most suitable one for this specific application. Since subject-dependent evaluation is usually easier than k-fold cross-validation in human-activity-recognition applications [54], the outstanding classifier according to the first mentioned criteria is examined in the later one. ML algorithms are referred to under the Scikit-learn framework in Python. Table 3 illustrates the parameters of each classifier during the experiments conducted here.

Performance of the examined ML algorithms is evaluated according to four metrics, namely the classification accuracy (Equation (3)); the F1-measure, which is the average of precision and recall of classification; (Equations (4) and (5)); execution time; and size on the disk.

(3) $$\mathrm{Accuracy}=\frac{TP}{TP+TN+FP+FN}$$

(4) $$\mathrm{Precision}=\frac{TP}{TP+FP}$$

(5) $$\mathrm{Recall}=\frac{TP}{TP+FN}$$

where TP represents the true-positive, TN is the true-negative, FP is the false-positive and FN is the false-negative classification rate. The best settings for each classifier are used in experiments after examining various training options. Experiments run on a computer machine with 10 GB RAM and 2.60 GHz i5 CPU.

### 3.2. Subject-Dependent Evaluation

**Figure 4:**
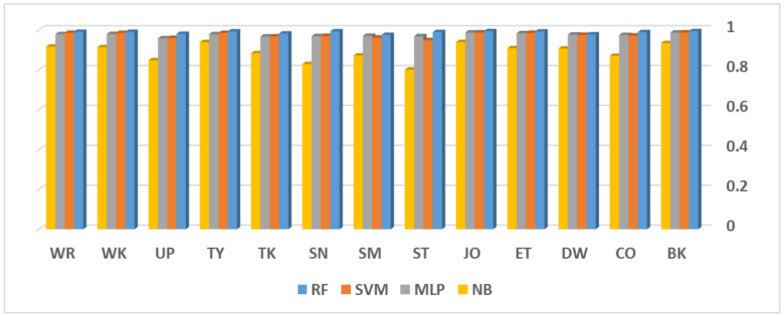
Average F-measure scores of the activities: Bike (BK), Coffee (CO), Downstairs (DW), Eat (ET), Jog (JO), Sit (ST), Smoke (SM), Stand (SN), Talk (TK), Type (TY), Upstairs (UP), Walk (WK) and Write (WR). Compared classifiers are evaluated under subject-dependent criteria.

The samples of each class are randomly separated, with 70% in the training and validation set and 30% in the testing set. The test samples are never introduced training any of examined classifiers, but samples of the same subject may appear in both training and testing sets. For impartial comparison, the simulation procedure was repeated by 10 independent runs, where each time, the same training/testing data are provided to each classifier. The average classification rates for activity recognition are presented in Figure 4.

**Figure 5:**
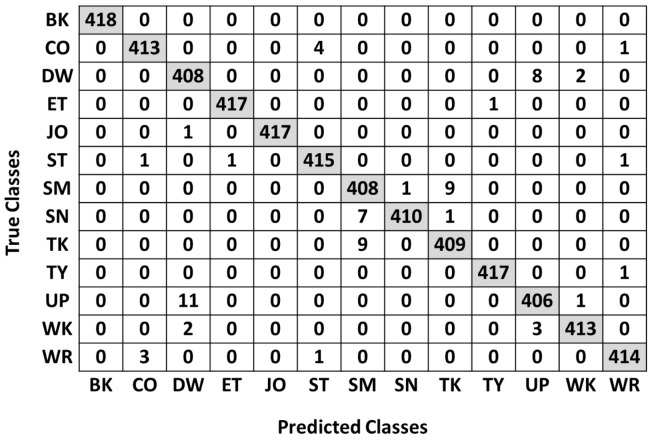
Confusion matrix for the RF classifier under subject-dependent evaluation criteria.

Figure 4 shows the average classification rates for different activities per classifier. RF has the highest rate for each activity. Biking, eating, jogging, sitting, typing, and writing activities are successfully recognized with a rate >99%. The activities walking downstairs, walking upstairs, and smoking are the least recognized by the RF classifier with a rate slightly less than 98%. Such behavior can be justified by reading the confusion matrix shown in Figure 5. On average, eight examples of walking downstairs were misclassified as walking upstairs, and vice versa for 11 examples of walking upstairs. Another notable conflict occurred for nine examples between smoking and giving a talk. It was noticed that conflicts occurred between very close activities, which is likely expected in such applications. However, the overall performance of the current model (employed sensors + preprocessing + features + classifier) is accepted, and it can be further improved by providing more training examples.

**Figure 6:**
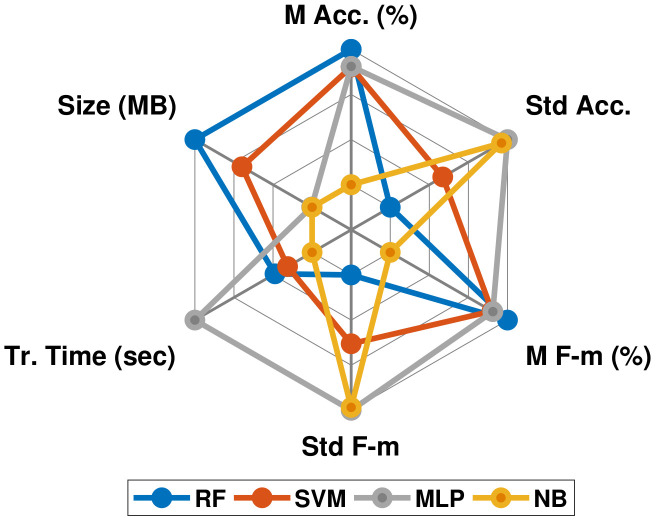
Radar plot for compared classifiers according to mean and standard deviation of accuracy (M Acc (%)) and (Std Acc), respectively; mean and standard deviation of F-measure (M F-m (%)) and (Std F-m), respectively; raining time in sec. (Tr. Time (sec)); and Size on disk in MB (Size (MB)).

Table 4 provides a summary of comparing different ML algorithms, as well as important implementation issues. On average, the accuracy (and F-measure) of RF reaches 98.72%, which exceeds the accuracy of each of SVM, MLP, and NB by 1.3%, 1.27%, and 11.1%, respectively. MLP takes a notably long training time of 90.41 s, while NB training occurred quickly at less than one second, and RF needed about 29.3 s to announce its decisions. RF occupies about 22.68 MB of the disk, which is the largest size, while NB needs only 0.046 MB space. To improve the readability of comparative results of all classifiers, Figure 6 presents an illustrative radar plot.

### 3.3. Stratified k-Fold Cross-Validation

In the experimental settings of collecting this dataset, a controlled protocol was performed by each of the 10 participants. Each participating subject performed the same set of activities within the same permitted time duration. Thus, by chance, for this particular dataset, 10-fold cross-validation implicitly involved the stratified 10-fold validation followed in Shoaib et al. [42]. Moreover, the common evaluation criterion for human activity recognition models, i.e., leave-one-subject-out, can also be applied via the 10-fold cross-validation for this particular dataset. The latter measure criteria are of interest where the dataset provides subject-independent evaluation, and hence it examines the model’s of generalization ability for newly introduced data. The average accuracy of the applied RF-based model here is equal to 92.54%.

## 4. Sensitivity Analysis for Model Parameters

**Figure 7:**
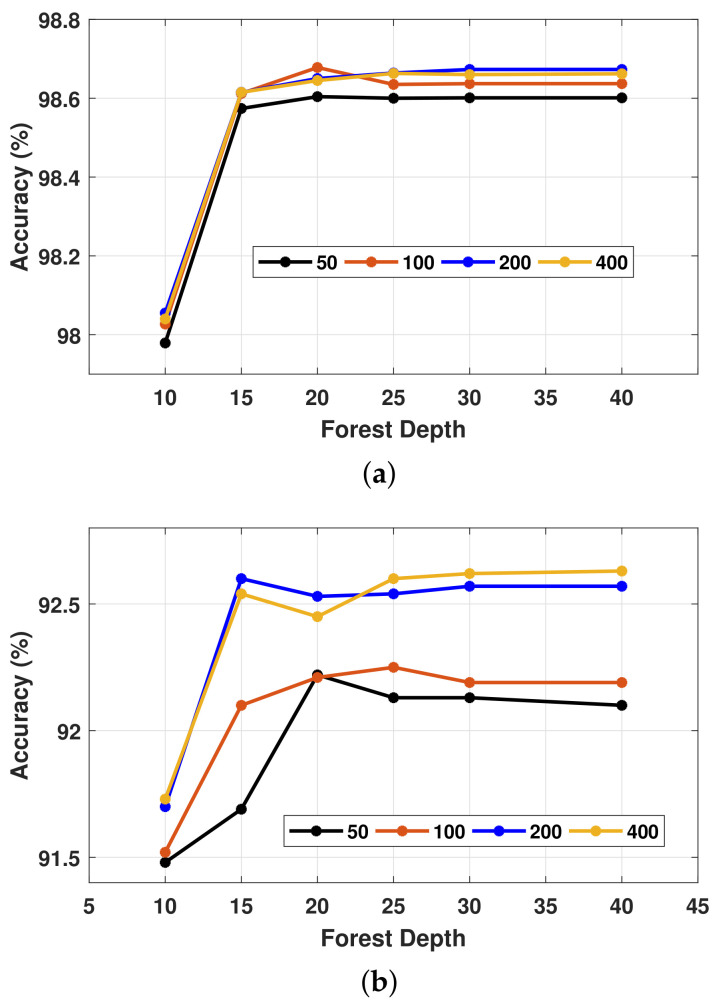
Model performance for different values of both forest size (50, 100, 200, 400) and forest depth (15, 20, 25, 30, 40) for (**a**) subject-dependent and (**b**) 10-fold cross validation criteria.

The performance of the RF algorithm is tremendously sensitive to both the number of decision trees (known as RF size) and the longest path from a tree head to the leaves (known as RF depth). For RF depth ≥15, with a suitable RF size ≥50, the applied RF-based model can provide notable recognition performance under subject-dependent evaluation measure; see Figure 7a. Moreover, increasing the RF size up to 400 trees has a slight improvement in the model accuracy. Conversely, under 10-fold cross-validation evaluation, the model accuracy grows by 1% when increasing both RF size and RF depth from (50, 10) to (15, 200); see Figure 7b. Moreover, increasing the RF size to 400, for example, will not enhance the model accuracy as much as the notable increment in processing time in this case. From Figure 7, we can conclude that with an RF depth between 15 and 25 and an RF size equal to 200, an efficient recognition model can be implemented for these kinds of IoHT systems that make use of sensory data from smartphones.

## 5. Comparison with Previous Studies

Different studies in the literature have addressed this dataset according to different evaluation measures. Table 5 provides the previous best recognition rates according to subject-dependent evaluation. Baldominos et al. [45] have tested shallow techniques against the deep CNN model. Only raw signals are used in 60 s segments. The ensemble of randomized decision trees (ET), with a set of handcrafted features, provides an average overall accuracy of 95.3%, while the accuracy of the CNN-based approach decreases to 85%. Stacked autoencoders provided better results than deep belief networks, where the accuracy reached 97.13% according to Alo et al. [46]. In a later study, besides raw activity signals, the magnitude vector and the vectors of pitch and roll angles were provided to deep networks in segments with a length of 2 s.

The proposed DL model was able to outperform the conventional classifiers such as support vector machines (SVM), Naive Bayes (NB), and linear discriminant analysis (LDA); however, the RF classifier was not included in this comparison. The current RF-based model presents the best recognition results among related studies. However, samples of the same person may appear in both the training and testing sets, but the experimental findings are still useful for seeking good models since registered data points occurred at different timestamps.

Moreover, the current model improves the recognition rates obtained by Shoaib et al. [42]. Table 6 shows the rates of each activity when stratified 10-fold cross-validation criteria are applied. Numerical values of Shoaib et al.’s model were computed from the confusion matrix in Figure 2c in [42]. The applied classifier was NB, but features were extracted from segments with a length of 5 s, and only accelerometer and gyroscope signals were used. Because of the suitable feature set used within the current model, the activities that directly depend on hand movement are well-recognized. The improvements in the rates of activities are as follows: having coffee (0.83 to 0.92), eating (0.89 to 0.99), smoking (0.82 to 0.95), giving a talk (0.86 to 0.97), typing (0.95 to 0.98), writing (0.89 to 0.97). For the other activities, the current model performs worse than or equal to Shoaib et al.’s model. In conclusion, the average overall accuracy is improved by 1.4%.

## 6. Applied Model Performance for WISDM Dataset

In this section, the validation of the applied framework is extended to the WISDM dataset [43]. It is one of the most addressed datasets in the HAR literature. WISDM v1 contains a total of 1,098,207 examples of activities that have been collected by 29 subjects. Six activities, namely walking (37.2%), jogging (29.2%), upstairs (12.0%), downstairs (10.2%), sitting (6.4%), and standing (5%), were registered via a smartphone in the front pants pocket (see Figure 1) of each subject. Walking and jogging activities were the most represented in this dataset. Activity signals were registered using the embedded accelerometer of the smartphone at a 20 Hz sampling rate. In the experimental settings, a window size of 10 s (according to the original study [43]) with 50% overlapping was applied to raw signals. The proposed feature set was generated for each activity segment, where the feature vector was 74 dimensions; since only the accelerometer signals are available, the RF classifier is called. Using the best settings, e.g., RF size and depth (200, 25), gave acceptable classification rates for this dataset. For 10-fold cross-validation criteria, the applied model gave an average accuracy of 94%, while for the subject-dependent evaluation (i.e., 70% training and 30% testing), the average accuracy reached 98.56%. This model performance regarding this dataset is comparable to many recent related studies in the literature, as summarized in Table 7.

Among the compared studies that appear in Table 7, using a window of 5 s for segments in [55] is more challenging than using longer segments, but a deep learning model was able to achieve 94.2% accuracy under 10-fold cross-validation. Moreover, an accuracy value of 98.85% was obtained in [56], but applying 95% overlapping when doing segmentation, and this is questionable in such a HAR study (i.e., overlapping usually ranges from 0 to 50%). In addition, for a 70%/30% split, using a more efficient RF such as (50, 20) gives an average accuracy of 98.34%, which is still close to the best performance obtained. However, under 10-fold cross-validation, using an RF with (50, 20) does not degrade the accuracy by more than 0.02%.

Summing up, the applied framework shows good performance for the WISDM v1 dataset under different evaluation criteria, while usually, only one of them is used in previous related studies. This model behavior reflects the robustness and suitability of both the feature set and the classifier algorithm for real-time HAR applications.

## 7. Discussion

The applied framework introduces one example of an IoHT system that is examined using two datasets with different settings. Shoaib’s dataset contains thirteen activities gathered by 10 subjects at a sampling rate of 50 Hz, while WISDM v1 has six activities collected by 29 subjects at a sampling rate of 20 Hz. Such a variety of activity signal resources constitutes a strong test for any proposed HAR model. Applying the different common evaluation criteria of HAR models in the same study is highly recommended to ensure its superiority. Later observation is missing in most studies in the literature.

More evidence is needed for the use of the dense production of deep learning models in the HAR field. Such models have thousands of parameters learned during training (tremendous computational load). However, they should at least outperform the conventional shallow approaches. Classical handcrafted features are meaningful and interoperable to a great extent, while the interpretation of most deep models, in particular in the HAR field, is still in its infancy.

In [46], the applied DL model required the help of extra inputs such as magnitude and pitch and roll signals, together with the raw 3D acceleration signals, in order to improve the performance. One the other hand, features extracted implicitly from DL models may need refinement via feature selection approaches in order to eliminate illusive features of classifiers. Recent studies such as [63] and others have emphasized the role of applying feature selection with DL models. On the other hand, the RF algorithm performs feature selection as one of the steps performed to achieve its classification result. One important observation is the degradation of accuracy when moving from the subject-dependent to 10-fold cross-validation criteria. For the WISDM v1 dataset, the misclassification is relatively high between upstairs and downstairs in comparison to other activities, in addition to the difficulty when applying 10-fold cross-validation (i.e., different subjects are used for training and testing). The later result has also been reported by different previous models such as [43,55,60], which cn probably be attributed to the sensor position on subjects’ bodies. A similar notation also holds for Shoaib’s dataset, where in Figure 5, the confusion matrix shows that the majority of false predictions take place between the activities of walking upstairs and walking downstairs.

## 8. Conclusions and Future Trends

In this work, an efficient model for an IoHT system is introduced through a set of carefully handcrafted features and a shallow classifier such as Random Forest for the dataset of Shoaib et al. [42]. Participants used to collect this dataset followed a specific protocol, which may be called a controlled environment. Similarly to related studies, using accelerometers and gyroscope sensors in smartphones is convenient for such applications. Moreover, inducing features (e.g., statistics of the roll angle vector and the angle of the x–component of body acceleration with a gravity vector) that depend on body kinematics (e.g., wrist and leg motion) improve the model performance. The presented model provides state-of-the-art results under both subject-dependent and 10-fold cross-validation criteria. Moreover, the current model performance was verified by another dataset, namely WISDM v1 [43] under both aforementioned evaluation criteria.

## Figures and Tables

**Table 1:** Dataset collection configuration.

Parameter	Information
# Subjects	10
# Activities	13
Total # Observations	1,170,000
Missing values	NO
Device	Two Samsung Galaxy S2 smartphones
Position on Body	Right pocket and right wrist
Sensors	Accelerometer, Linear Accelerometer, Gyroscope and Magnetometer
Frequency	50 Hz

**Table 2:** Dataset activities.

Activity	Abbreviation	Duration (min)
Biking	BK	3
Having Coffee	CO	5
Walking Downstairs	DS	3
Eating	ET	5
Jogging	JO	3
Sitting	ST	3
Smoking	SM	5
Standing	SN	3
Giving a Talk	TK	5
Typing	TP	5
Walking Upstairs	UP	3
Walking	WK	3
Writing	WR	5

**Table 3:** Classifiers settings and parameter values.

Classifier	Function Call	Settings and Parameters
RF	*RandomForestClassifier()*	# estimators = 200, max. depth = 25, min. samples split = 2
MLP	*MLPClassifier()*	solver: quasi-Newton method, # hidden neurons = 75, activation function: tanh, max. # iterations = 1000, momentum = 0.9, initial learning rate is 0.01, validation ratio = 15%
SVM	*svm.SVC()*	kernel: radial basis function, polynomial degree is 3
NB	*GaussianNB()*	μ and σ parameters of Gaussian distribution are estimated using maximum likelihood

**Table 4:** Performance of compared classifiers for subject-dependent evaluation.

	Accuracy		F-Measure		Training Time (sec)	Size on Disk (MB)
RF	Mean	98.72	Mean	98.72	29.3	22.683
	Std	0.1015	Std	0.1015		
SVM	Mean	97.43	Mean	97.42	19.69	13.593
	Std	0.2279	Std	0.2398		
MLP	Mean	97.47	Mean	97.49	90.41	0.143
	Std	0.3837	Std	0.3736		
NB	Mean	88.82	Mean	88.87	1	0.046
	Std	0.3693	Std	0.3677		

**Table 5:** Recognition rates of each activity for different models under subject-dependent validation criteria. ET: ensemble of randomized trees, FC: fully connected layer, AE: autoencoders, and DBN: deep belief networks.

Reference	Input Signals	Segment Length (s)	Feature Extraction	Classifier	Accuracy (%)
Baldominos et al. [45]	Raw signals	60	Handcrafted	ET	95.3
Baldominos et al. [45]	Raw signals	60	CNN hidden layers	FC layer	85
Alo et al. [46]	Raw signals, magnitude vector, pitch and roll vectors	2	Sparse AE layers	FC layer	97.13
Alo et al. [46]	Raw signals, magnitude vector, pitch and roll vectors	2	DBN hidden layers	DBN output layer	91.57
Current model	Raw signals	2.56	Handcrafted	RF	98.7

**Table 6:** Recognition rates of each activity for different models under 10-fold cross validation criteria.

Ref.	BK	CO	DW	ET	JO	ST	SM	SN	TK	TY	UP	WK	WR	Accuracy (%)
Shoaib et al. [42]	0.99	0.83	0.98	0.89	1	0.90	0.82	0.92	0.86	0.95	0.96	0.85	0.89	91.2
Current model	0.99	0.92	0.91	0.99	0.99	0.76	0.95	0.94	0.97	0.98	0.83	0.80	0.97	92.54

**Table 7:** Applied model results for WISDM dataset. MLP: multi-layer perceptron. LR: logistic regression. Stat. Feat.: statistical features. Att. M.: attention mechanism. R. B.: residual block. LSTM: Long short-term memory.

Evaluation	Reference	Segment Length (s)	Feature Extraction	Classifier	Accuracy (%)
10-fold cross validation	Kwapisz et al. [43]	10	Handcrafted	MLP	91.7
	Garcia-Ceja et al. [55]	5	CNN	FC layer	94.2
	Catal et al. [57]	10	Handcrafted	Ensemble of (LR, MLP, j48)	91.62
	Ignatov [58]	10	CNN + Stat. Feat.	FC layer	93.32
	Current model	10	Handcrafted	RF	94
70%/30% split	Gao et al. [56]	10	CNN + Att. M.	FC layer	98.85
	Suwannarat et al. [59]	8	CNN	FC layer	95
	Abdel-Basset et al. [60]	10	CNN + R. B. + LSTM + Att. M.	MLP	98.90
	Zhang et al. [61]	11.2	CNN	FC layers	96.4
	Zhang et al. [62]	10	CNN + Att.	FC layer	96.4
	Current model	10	Handcrafted	RF	98.56

## Data Availability

The data are publicly available as described in the main text.
